# Quality improvement initiative to decrease severe intraventricular hemorrhage rates in preterm infants by implementation of a care bundle

**DOI:** 10.1038/s41372-025-02274-5

**Published:** 2025-03-27

**Authors:** Samantha D. Peltola, Uduak S. Akpan, Dmitry Tumin, Penni Huffman

**Affiliations:** 1 College of Nursing, University of South Alabama, Mobile, AL USA; 2 Brody School of Medicine at East Carolina University, Greenville, NC USA

**Keywords:** Preventive medicine, Health care

## Abstract

**Objective:**

To decrease the incidence of severe Intraventricular Hemorrhage (sIVH) rates in preterm infants in a Neonatal Intensive Care Unit (NICU) to 10% over eight months.

**Population:**

All infants born less than 30 gestational weeks.

**Methods:**

Targeted interventions focus on the first 72 h of life and include minimizing disturbance in cerebral blood flow and minimal stimulation. Data were collected from a review of electronic medical records from September 2023 – April 2024.

**Outcome measures:**

A decrease in sIVH (grade III-IV), an increase in head-of-bed elevation rate, and an evaluation of pedal/pelvic edema due to head-of-bed elevation.

**Results:**

The rate of sIVH decreased over the eight-month implementation period from 24.4% in the baseline period to 9.3% in the intervention period.

**Conclusion:**

Standardization of care using a sIVH prevention bundle was instrumental in the success of this project.

## Introduction

### Problem description

Premature infants in the neonatal intensive care unit (NICU) have an increased risk of developing an intraventricular hemorrhage (IVH). Approximately 50% of infants born in the United States that are less than 35 gestational weeks or have a birth weight of less than 1500 grams are diagnosed with an IVH [1]. IVH is the leading cause of disability and mortality among preterm patients, making it a significant quality issue in healthcare [2]. It has been linked to periventricular white matter injury, periventricular hemorrhagic infarction, and dispersed injury to the infant’s brain [1]. These injuries can result in posthemorrhagic hydrocephalus, cerebral palsy, severe cognitive impairment, and hearing/vision loss [3].

Grading systems are in place to define the significance of IVH using cranial ultrasound and evaluation by a radiologist. A grade I IVH is a hemorrhage confined within the subependymal germinal matrix, grade II hemorrhages extend into the lateral ventricles but do not cause ventricular dilation and occupy less than 50% of the ventricle, grade III hemorrhages occupy more than 50% of the ventricle and/or have associated ventricular dilation, and grade IV hemorrhages are associated with bleeding into the surrounding parenchyma. Grade I and II hemorrhages are considered mild while grades III and IV are considered severe [3].

In 2021 and 2022, the severe intraventricular hemorrhage (sIVH) rates at an eastern North Carolina academic medical center’s NICU were 10.8% and 22.8%, respectively. In comparison, the median sIVH rates in the United States in 2021, 2022, and 2023 were 9.5%, 9.6%, and 6.7%, respectively [4]. A process change was needed within our facility to decrease the incidence of sIVH. A positive patient outcome was a goal worth following and implementation of evidence-based practice assisted in achievement.

### Available knowledge

After a comprehensive analysis of relevant published articles on IVH prevention, sIVH has been addressed in multiple different settings with the implementation of an IVH prevention bundle. While there are many factors involved in IVH prevention, care bundles have proven effective in lowering the risk of developing any degree of IVH, cystic periventricular leukomalacia, and/or mortality [5]. Each intervention in these bundles provides a reduced risk of IVH but when all interventions are provided together, better outcomes are seen.

Maintaining the patient’s head in a midline position and elevating the head of the bed by approximately 15–30 degrees for the first 72 h of life optimizes cerebral venous drainage through the internal jugular veins and facilitates hydrostatic cerebral venous outflow in premature infants. Turning the head toward either side can cause ipsilateral occlusion of this drainage system which leads to alterations in blood flow, increased cerebral blood volume, and increased intracranial pressure, all of which can increase the risk of IVH [3].

Increased noxious stimuli can lead to disturbances in cerebral blood flow, increasing the risk for IVH. Preventing loud noises and aggressive handling, covering the infant’s eyes when bright lights are necessary, and adhering to specified care times can assist staff in providing a minimal stimulation environment [6].

Avoidance of endotracheal tube suctioning whenever possible is another intervention identified in the literature that can aid in decreasing the risk of IVH. Aspiration of secretions during suctioning can cause an alteration in blood pressure, intracranial pressure, and cerebral blood flow. It is often a practice in NICUs to routinely suction the endotracheal tube before the collection of blood gasses. If possible, this should be avoided during the first 72 h of life to avoid the shift in intracranial pressure, thus decreasing the risk of IVH development [7].

### Rationale

#### Quality improvement model

An IVH quality improvement initiative was implemented in September of 2023 for all infants born less than 30 gestational weeks. This initiative was implemented in a level IV NICU at an academic medical center to decrease the sIVH rates. The Plan-Do-Study-Act method was used, and the first two cycles have been evaluated [8].

#### Evidence-based practice model

Johns Hopkins Nursing Evidence-Based Practice Model was used to implement evidence-based practice in this clinical setting. This model utilizes a three-step process: practice question, evidence, and translation [9].

### Practice question

In infants born less than 30 gestational weeks, how do IVH prevention bundles, compared with traditional hospital care, affect the incidence of IVH?

### Evidence

A literature review, appraisal, and rating of the evidence was performed. It was determined that care bundles have proven effective in lowering the risk of developing any degree of IVH, cystic periventricular leukomalacia, and/or mortality [5]. Evidence-based practice and current care bundles were further evaluated and prepped for implementation.

### Translation

In this stage, feasibility was identified and a plan was developed. The IVH bundle was implemented and evaluated. Results were then disseminated to the healthcare team.

#### Nursing theory

Martha Rogers’ Science of Unitary Human Beings Nursing Theory focuses on human beings as a whole and describes patients as being one with their environment [10]. Two key points are identified within this theory: the science of nursing, which is based on knowledge and research, and the art of nursing, which involves creatively using the science within nursing to improve the patient’s quality of life.

Our IVH prevention bundle aligns with this nursing theory because the overall goal of the project was to improve patient’s quality of life. It was focused on changing aspects of the patient’s environment and care to improve patient outcomes. Our project used research, science, and the environment to help achieve this goal.

### Specific aims

The overall purpose of our project was to decrease the incidence of sIVH in preterm infants in the NICU of an academic medical center with the implementation of an IVH prevention bundle. Our facility’s most recent IVH rates were 10.8% and 22.8% in 2021 and 2022, respectively. The goal outcome, referring to the desired result, was to decrease the sIVH rate to 10%. This theoretically should have minimized the detrimental outcomes that often come along with this diagnosis. The decrease in IVH should improve patients’ overall quality of life.

## Methods

### Context

Participants were chosen based on gestational age at birth. The inclusion criteria were all infants born less than 30 gestational weeks. Exclusion criteria were all infants born greater than 29 6/7 gestational weeks. Based on the 2021 admission rates, it was estimated that approximately 80 infants would be included in the project over the eight-month implementation period.

The project was conducted at an eastern North Carolina academic medical center’s NICU. This unit houses a 50-bed level IV NICU and a 21-bed intermediate care nursery. Annually, there are approximately 1100 admissions and 3500 deliveries at the high-risk obstetrical delivery service.

The unit is staffed by registered nurses, certified nursing assistants, respiratory therapists, neonatal nurse practitioners, physician assistants, fellow/resident physicians, and board-certified neonatologists. High-risk deliveries are attended by registered nurses, respiratory therapists, and at least one provider. All infants born less than 32 gestational weeks fall into the golden hour criteria which includes focused medical interventions to stabilize these infants during their most vulnerable time. Each of these infants receives head ultrasounds at 5–7 days of life and then again at 4–6 weeks of life. Any infant born less than 30 gestational weeks and/or less than 1000 grams also receives a head ultrasound at 6 h of life. Before the implementation of this bundle, IVH prevention measures included maintaining the head in the midline position and avoiding fluid boluses.

### Interventions

An IVH prevention bundle (see Appendix A) was implemented for all patients born less than 30 gestational weeks who were admitted to the NICU. Project implementation was focused on all individuals involved in the patient’s care during the first 72 h of life. These individuals included providers (neonatal nurse practitioners, physician assistants, fellows/resident physicians, and attending physicians), nursing staff (staff/floor nurses, nurse managers, and the nurse educator), respiratory therapists, and the patient’s primary caregivers/guardians.

This bundle included multiple evidence-based interventions that have proven to be effective in the reduction of sIVH in this population. These interventions focused on minimal stimulation, minimizing disturbances in cerebral blood flow, and parental involvement. Bundle initiation began on admission to the NICU and was continued for the first 72 h of life. The initial timeline for project implementation was approximately four months but this timeline was extended to eight months after it was discovered a longer implementation period was needed.

Training individuals involved was performed regularly before implementation and discussed during unit counsel/staff meetings. The bundle was disseminated to all staff via the nurse manager and unit director before initiation.

### Study of the interventions

The overall project goal was evaluated by assessing the sIVH rate after project implementation. Evaluation of head ultrasound reports was completed to determine if sIVHs were present after bundle implementation. A baseline period was established by performing retrospective data collection and analysis on the same population in the four months before implementation. The sIVH rates in the baseline and intervention periods were compared. Statistical process control charts were used to establish if the improvements made were enough to show a center-line change.

Chart reviews were completed to assess the process measure (elevation of the head-of-bed in the intervention group) and the balancing measure (pedal/pelvic edema in baseline and intervention group due to elevation of the head-of-bed).

The literature did not present a specific existing valid and reliable tool used to evaluate project effectiveness, therefore, evaluation of project effectiveness was determined by assessment of data collection results.

### Measures

1. Outcome Measure: We tracked the rates of IVH in premature neonates born less than 30 gestational weeks with a goal of a 14.4% reduction. The denominator was all neonates born less than 30 gestational weeks and admitted to the NICU, while the numerator was neonates with sIVH noted within the first week of life.
2. Process Measure: We measured the percentage of neonates born less than 30 gestational weeks whose head-of-bed was elevated, with our goal being 80%. The numerator for this measure was patients whose head-of-bed was elevated (15–30 degrees) in the first 72 h of life and the denominator was all neonates born at 30 gestational weeks and admitted to the NICU.
3. Balancing Measure: To ensure that our patients were not experiencing adverse events due to the interventions, we selected the rates of lower body edema as our balancing measure with the goal of less than 30%. The numerator for this measure was patients with swelling of any lower body area, including sacrum, groin, legs, or feet and the denominator was the same as for other measures.

These measures were considered quantifiable techniques used to track progress towards the goal outcome.

### Analysis

We manually extracted data from electronic medical records (EMR; Epic, Verona, WI). We collected baseline data over four months, from May 2023 to August 2023. The intervention period occurred over four months, from September 2023 to December 2023, and we collected data for a further four months, from January 2024 to April 2024, to ensure the sustainability of the improvement we observed.

We tracked measures for patients in cohorts of eight patients each to ensure uniformity of the groups, using statistical process control charts (QI Macros, Denver, CO: KnowWare International), with special cause variation defined using previously described rules [11]. A chi-square test was performed to compare data between the baseline and intervention groups. A Mann-Whitney (Wilcoxon) test for unpaired data was also performed to evaluate if the changes seen were due to gestational age or weight differences between groups. A *p-*value of < 0.05 was considered statistically significant. Data analysis was performed using IBM SPSS Statistics Version 29 and Excel. The project was described using the SQUIRE 2.0 guidelines for QI reporting [12].

### Ethical considerations

The Institutional Review Board (IRB) at our institution determined this project was not human subject research. Approval was obtained from the University of South Alabama’s IRB. This quality improvement initiative was performed in accordance with the Declaration of Helsinki.

## Results

During the study period, 122 neonates met the inclusion criteria (44 in the baseline group from May 2023 to August 2023 and 78 in the intervention group from September 2023 to April 2024). Six of these neonates (three in the baseline group and three in the intervention group) died before receiving the head ultrasound at five to seven days of life and were removed from the data. A Mann-Whitney (Wilcoxon) test for unpaired data was performed to assess the gestational age and birth weight differences between groups. There were no significant differences in gestational age (*p* = 0.497) or weight (*p* = 0.255). The characteristics of the cohorts are summarized in Table 1.

**Table 1:** Patient characteristics in baseline and intervention periods.

Variable	Baseline Period (*N* = 41) *N* (%) or Median (IQR)	Intervention Period (*N* = 75) *N* (%) or Median (IQR)	*P*
Gestational age (wk)	26.7 (22–29.6)	27.1 (22.1–29.6)	0.497
Birth weight (g)	914 (410–1695)	988 (380–1760)	0.255
sIVH	10 (24.4)	7 (9.3)	<0.05
Head-of-bed elevation	N/A	71 (95)	
Pedal/Pelvic edema	10 (24.4)	16 (21)	

A chi-square test of independence was calculated comparing the frequency of sIVH for patients in the baseline group and patients in the intervention group. A significant interaction was found (*x*^2^(1) = 4.805, *p* <0.05). Patients were more likely to develop a sIVH in the baseline group (24.4%) than they were in the intervention group (9.3%).

**Fig. 1:**
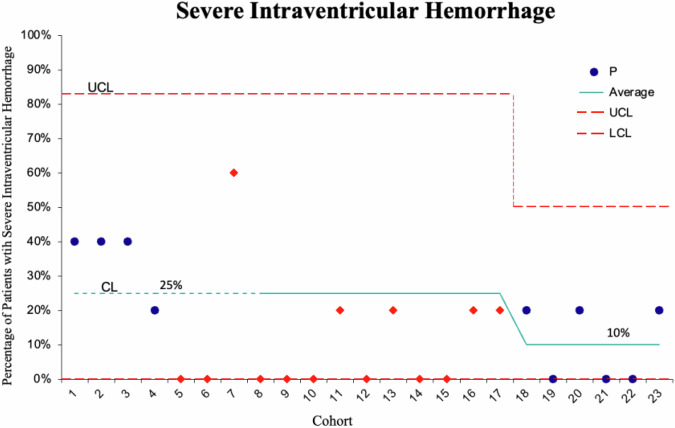
Severe Intraventricular Hemorrhage Rates. Outcome Measure. A run chart depicting the percentage of patients with sIVH at one week of life in the baseline and intervention groups.

When assessing the incidence of sIVH, the improvements made were significant enough to show a change in the center line, which represents the mean of the values. This is significant because it indicates that there was a shift in the average value being evaluated, demonstrating a clear improvement in the process. In the baseline period, the rate of sIVH was 25%, decreasing to 10% by the end of the project, reflecting a 60% decrease in the baseline rate and achieving our project goal (see Fig. 1). The absolute decrease in sIVH was 15.1% but the relative decrease was 24.4–9.3/24.4 = 61.9%.

**Fig. 2:**
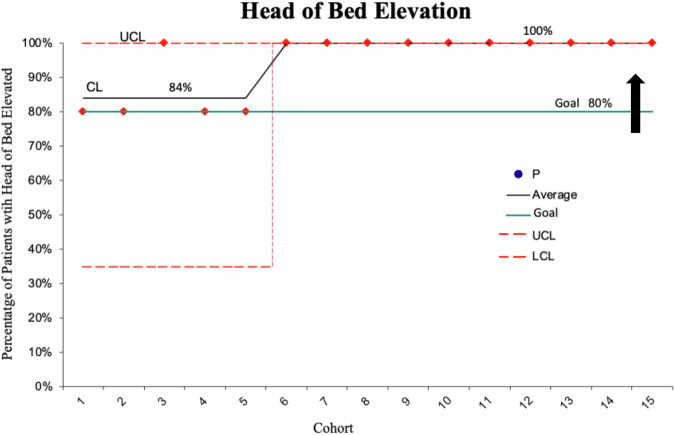
Head-of-Bed Elevation Rates. Process Measure. A run chart depicting the percentage of patients with elevated head-of-bed in the intervention group.

**Fig. 3:**
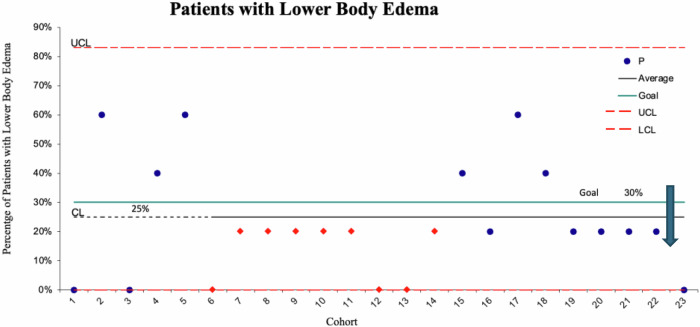
Lower Body Edema Rates. Balancing Measure. A run chart depicting the percentage of patients with pedal/pelvic edema due to head-of-bed elevation in the baseline and intervention groups.

Regarding the process measure (head-of-bed elevation), the goal of 80% in the intervention group was met as the center line is at 84% (see Fig. 2). The goal associated with the balancing measure (less than 30% of patients will experience pedal/pelvic edema) was also met as the center line is at 25% (see Fig. 3).

## Discussion

### Summary

The goal of decreasing the sIVH rate to 10% with the use of an IVH prevention bundle was achieved as the incidence was 9.3% in the intervention group. In addition, head-of-bed elevation was maintained at a rate of 95%, and less than 30% of patients experienced pedal/pelvic edema as a result.

The IVH prevention bundle checklist allowed for a smooth transition into nursing and provider practice. For all infants that met inclusion criteria, an admission order was placed in the electronic medical record to implement the bundle. Laminated cards were placed on the patient’s bed to remind staff to implement the checklist. Overall, the main strength of the project was the ease of implementation into practice. There were no significant changes that caused major hesitation from staff.

### Interpretations

In comparison to quality improvement initiatives performed by other centers to decrease sIVH rates, our bundle is very similar. A recent quality improvement project performed at a children’s teaching hospital [13] implemented a bundle focused on midline positioning, two-person care, slow infusion/aspiration from central lines, coordinated care every six hours, minimal stimulation, and thermoregulatory practices. This center’s sIVH rate decreased from 9.8% to 2.4% after bundle implementation.

Another care bundle implemented in two Dutch tertiary NICUs [5] focused on maintaining the head in the midline position, elevating the head-of-bed, avoiding sudden elevation of the legs, and preventing rapid flushing or withdrawing of blood/fluid from central lines. This center focused on the development of IVH within the first 72 h after birth and compared the control group (pre-intervention) and an intervention group. Their composite outcome of new or progressive severe germinal matrix IVH within the first 72 h, in-hospital death, or cystic periventricular leukomalacia decreased from 20% in the control group to 13% in the intervention group.

Our IVH prevention bundle is very similar to both centers’, however, ours does not include two-person care and has a category focused on parental involvement. Whether these differences played a factor in the sIVH rate is difficult to tell, given each intervention in these bundles provides a reduced risk of IVH, but when all interventions are provided together, better outcomes are seen.

In agreement with other centers’ reports that the use of an IVH prevention bundle is effective in lowering the risk of developing any degree of IVH, we noticed a decrease in sIVH rates in our NICU within the project period. The bundle focused on nursing-specific care with modifications to the usual care practices in extremely premature infants. Thorough education and a bundle checklist for nursing staff to follow eased the process of implementation. For a quality improvement project to be successful and sustainable, a multi-disciplinary effort is critical. Hence, modifications were made to the admission orders in the electronic medical record to include an IVH prevention bundle order for the providers to use. The main strength of this project is that it demonstrates the ease of implementation of a care bundle. Ensuring the education of all team members and guaranteeing that the burden did not fall solely on one discipline was essential to the project’s success.

It was noted that when assessing for pedal/pelvic edema in the intervention group, many patients had edema documented within the first 72 h of life. This was an unexpected finding which led to edema analysis in the baseline group as well as the intervention group. As depicted in Fig. 3, a higher incidence of edema was noted in the baseline group when compared to the intervention group, leading the readers to believe that head-of-bed elevation did not increase the incidence of pedal/pelvic edema.

There were no associated costs or strategic trade-offs associated with this project.

### Limitations

Initially, a limited implementation period was identified as the main limitation of this quality improvement initiative. Originally, data collection and analysis took place between September of 2023 and December of 2023. At this time, the patients were split into cohorts to display them adequately on control charts. One can argue that a center-line change was not initially observed because the incidence was so low that going from 1-2 per cohort to 0-1 per cohort was not a big enough change, or in the case of such small numbers, a larger cohort or a longer period was needed. Due to this noted limitation, data collection and analysis were extended another four months, allowing for a longer implementation period and more time to see a center-line change on the control chart.

There have been no factors identified that might have limited internal validity such as confounding, bias, or imprecision in the design, methods, measurement, or analysis.

## Conclusions

The diagnosis of a sIVH can be detrimental. Reducing the incidence of this diagnosis in the NICU can significantly improve patient outcomes. Implementing interventions that can make this possible and sustaining this improvement are the first steps toward improving the quality of care provided to this fragile population. This quality improvement initiative demonstrated that the implementation of an IVH prevention bundle decreased the incidence of sIVH in infants born less than 30 gestational weeks.

## Supplementary information

Appendix A

## Data Availability

The datasets generated during and/or analyzed during the current study are available from the corresponding author on reasonable request.
