# Supplementary figures and images for: Quality improvement initiative to decrease severe intraventricular hemorrhage rates in preterm infants by implementation of a care bundle
Source: J Perinatol. 2025 Mar 27;45(8):1152–7. doi: 10.1038/s41372-025-02274-5 (PMC12367532; doi:10.1038/s41372-025-02274-5)

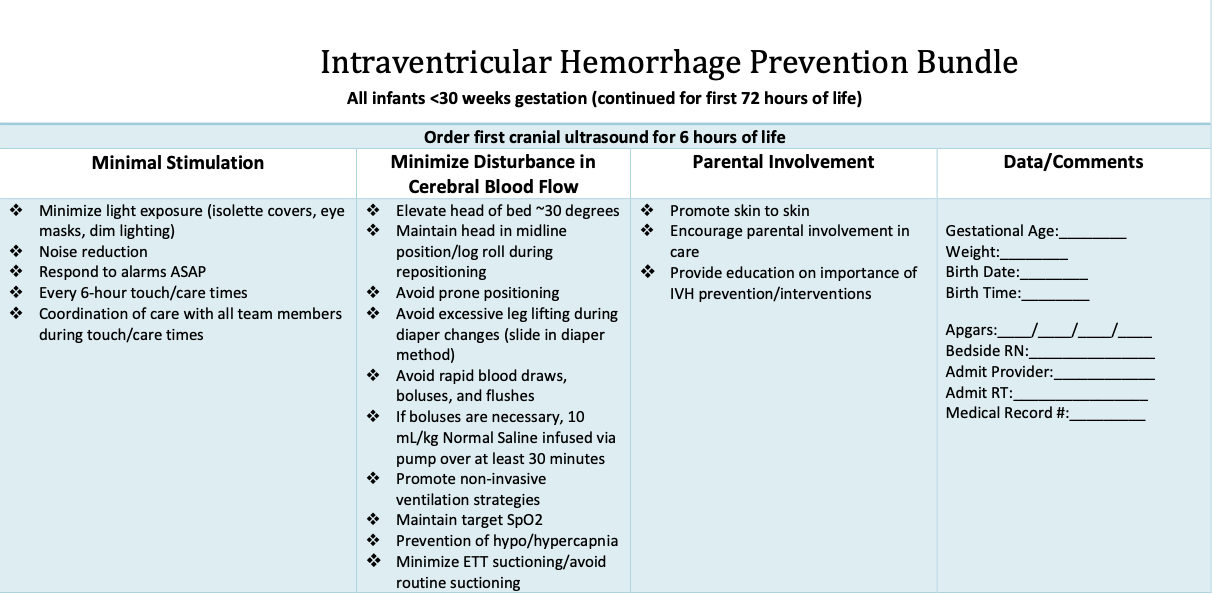

Supplement: Supplementary file 1 — Appendix A [file 41372_2025_2274_MOESM1_ESM.tif]
